# Supplementary figures and images for: Benchmarking predictions of MHC class I restricted T cell epitopes in a comprehensively studied model system
Source: PLoS Comput Biol. 2020 May 26;16(5):e1007757. doi: 10.1371/journal.pcbi.1007757 (PMC7274474; doi:10.1371/journal.pcbi.1007757)

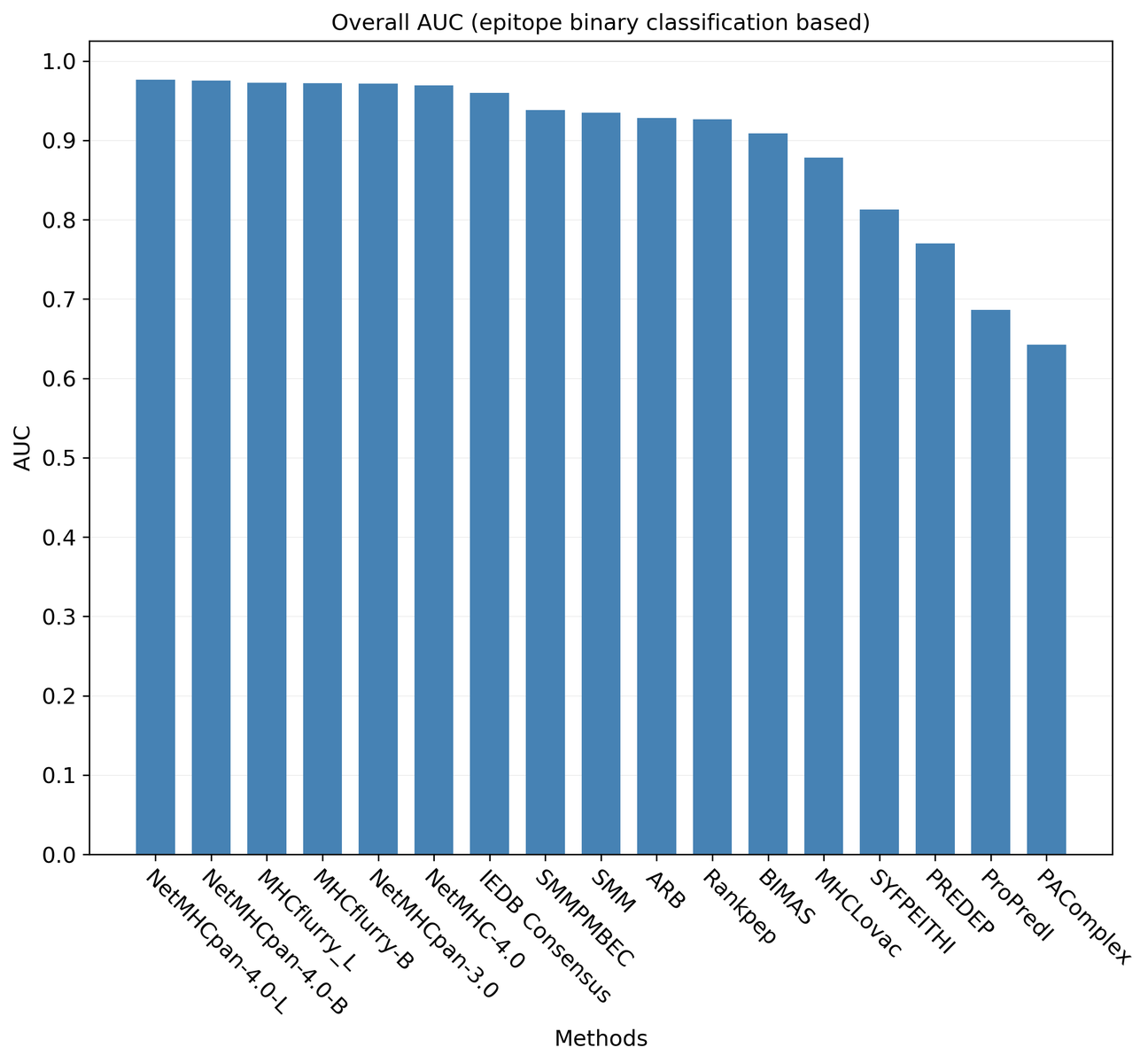

Supplement: S1 Fig — (TIF) [file pcbi.1007757.s002.tif]

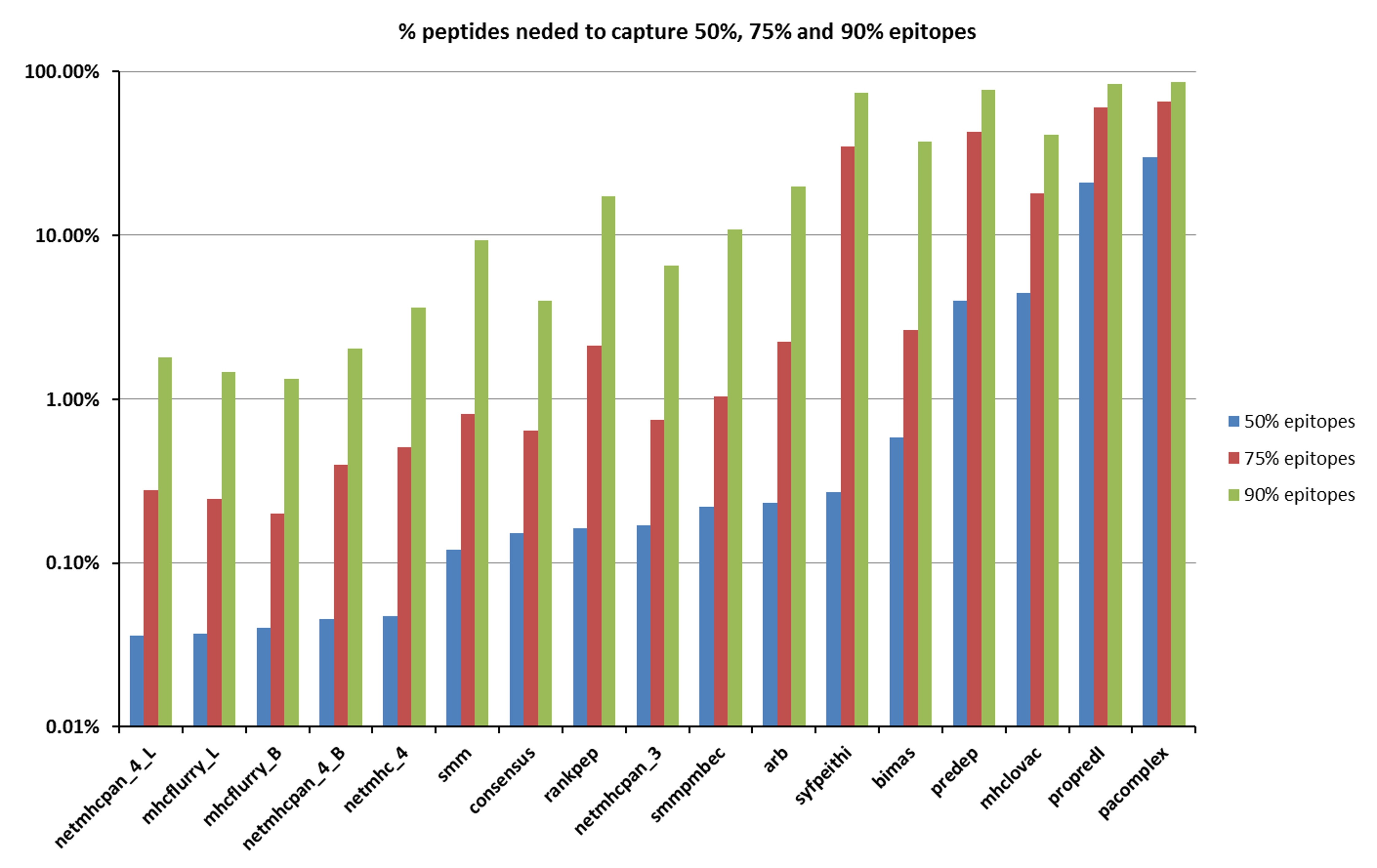

Supplement: S2 Fig — (TIF) [file pcbi.1007757.s003.tif]

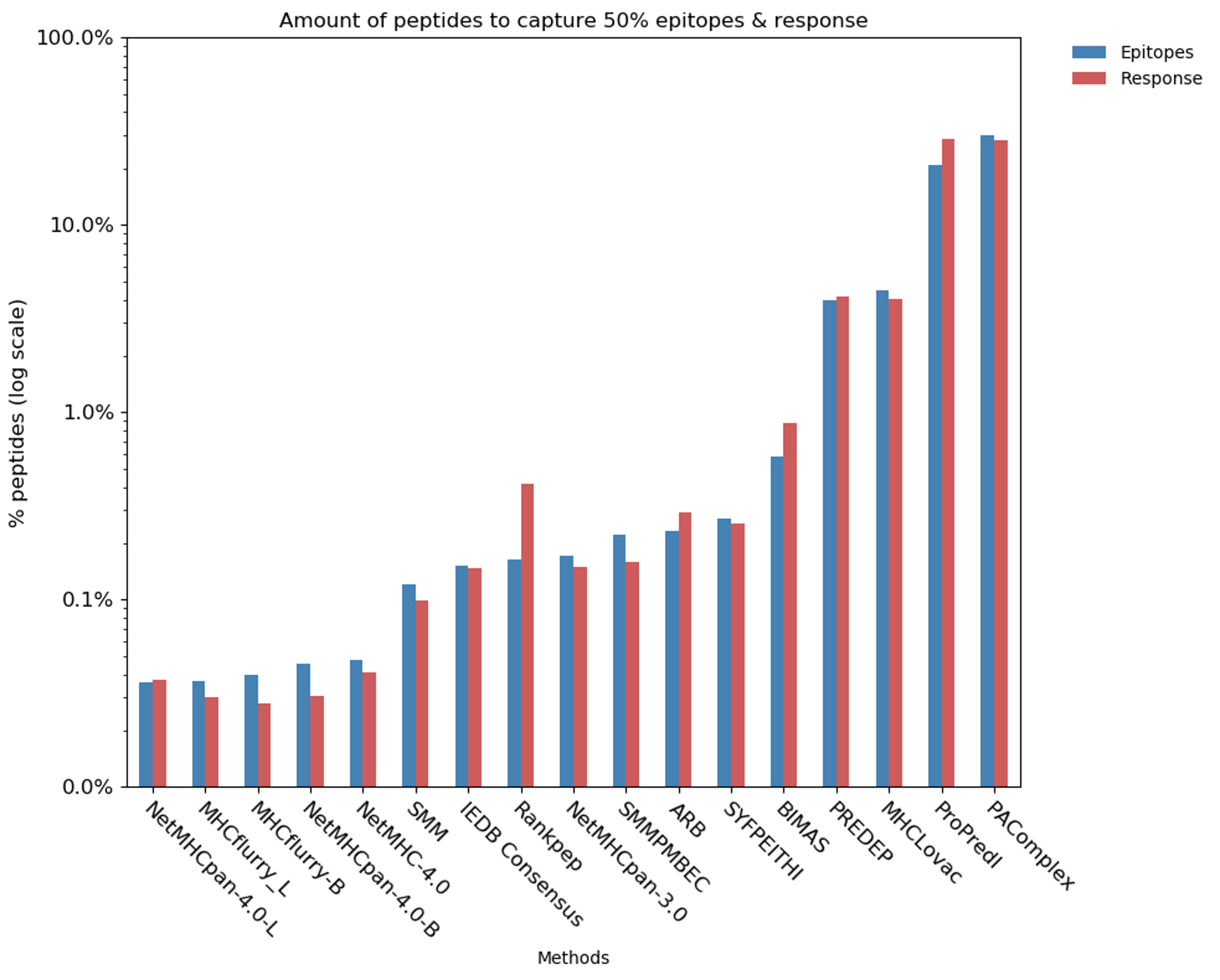

Supplement: S3 Fig — (TIF) [file pcbi.1007757.s004.tif]

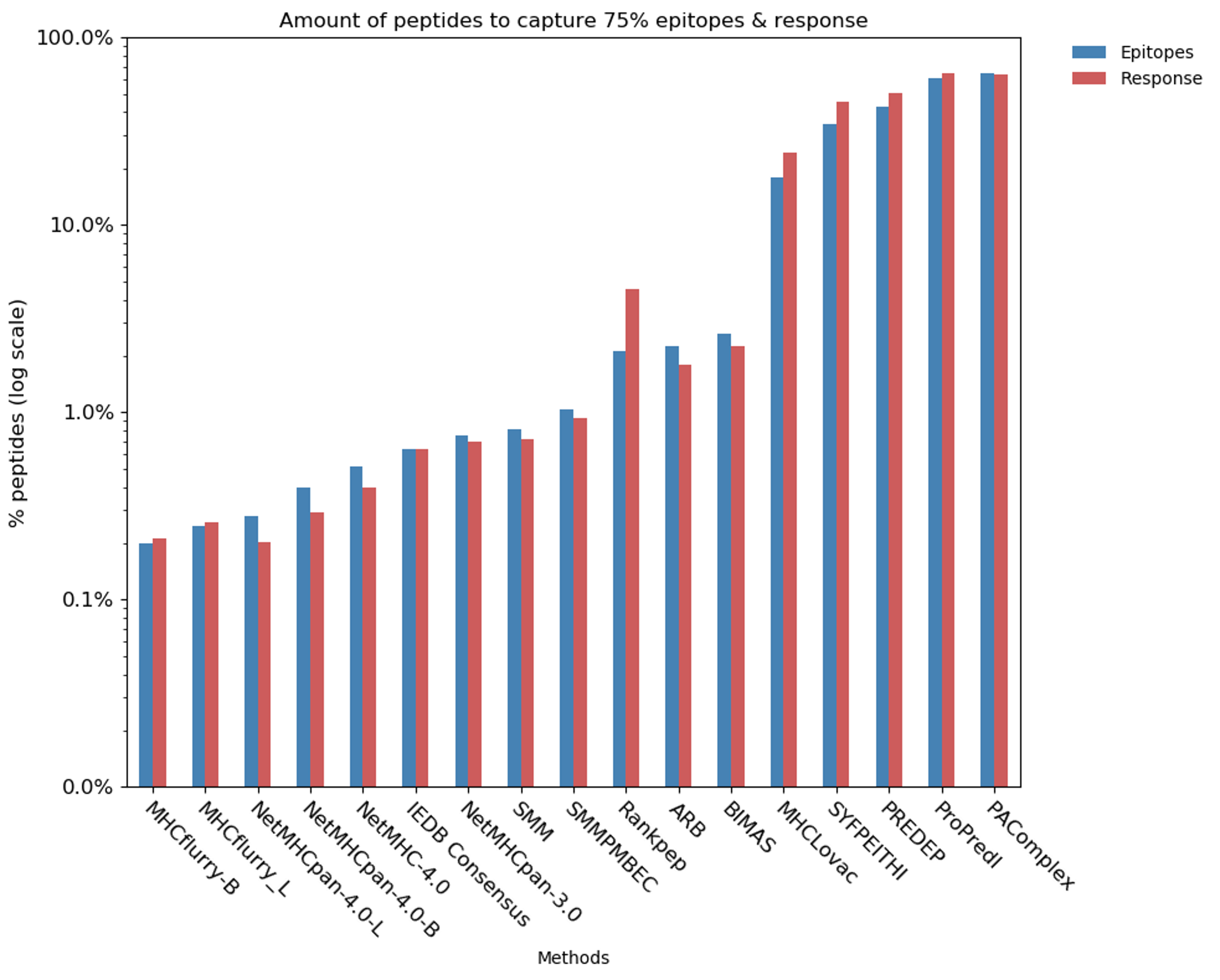

Supplement: S4 Fig — (TIF) [file pcbi.1007757.s005.tif]

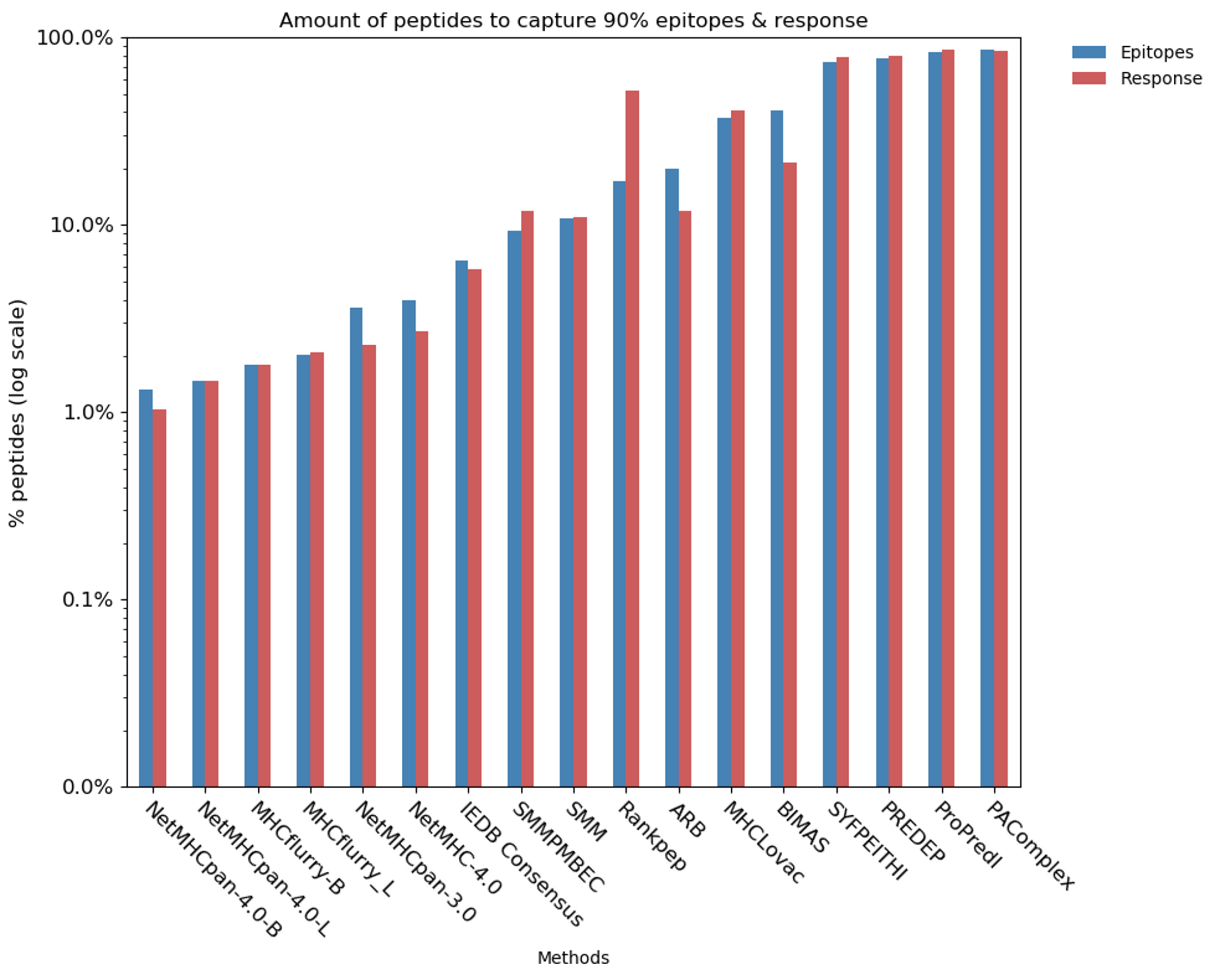

Supplement: S5 Fig — (TIF) [file pcbi.1007757.s006.tif]

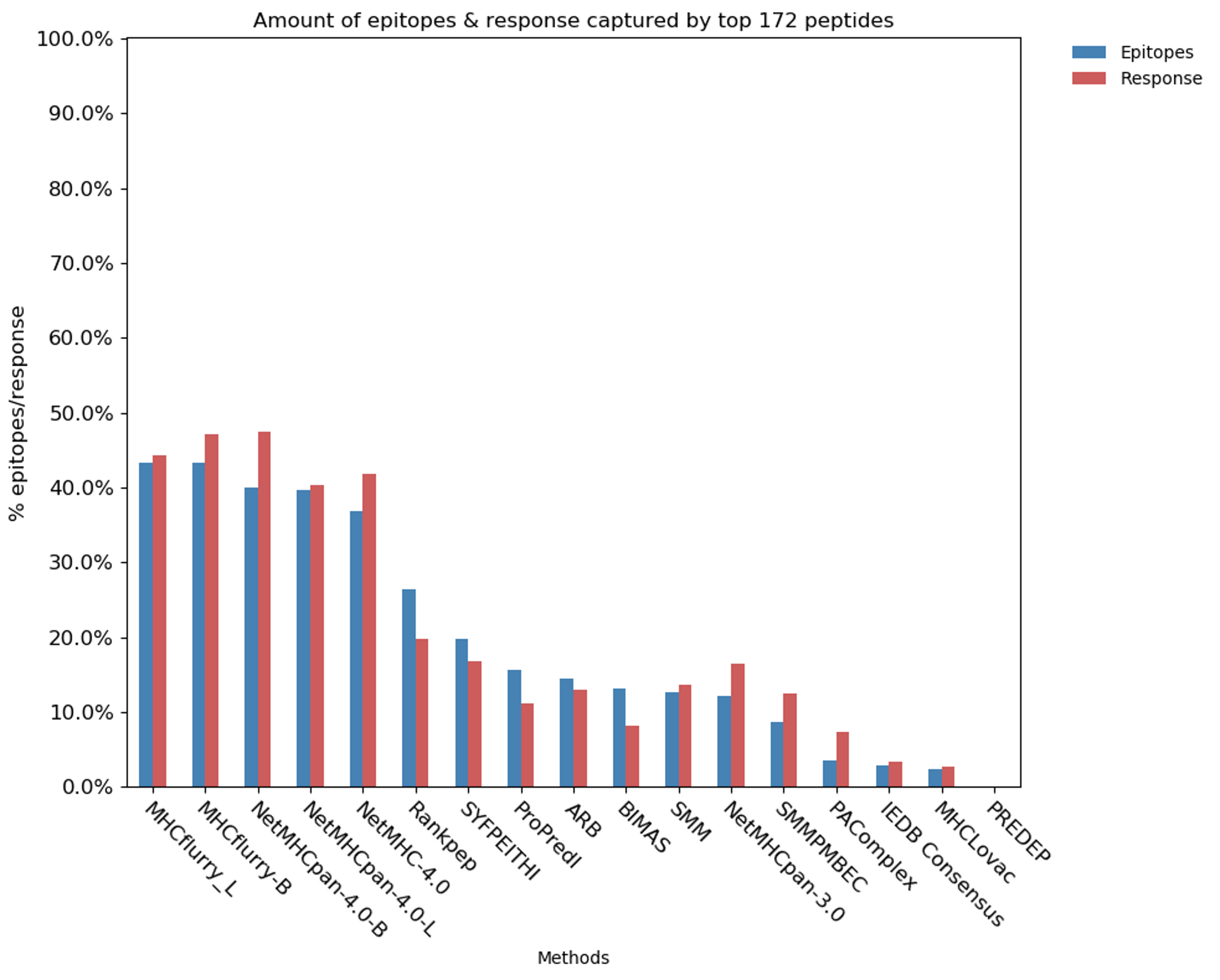

Supplement: S6 Fig — (TIF) [file pcbi.1007757.s007.tif]

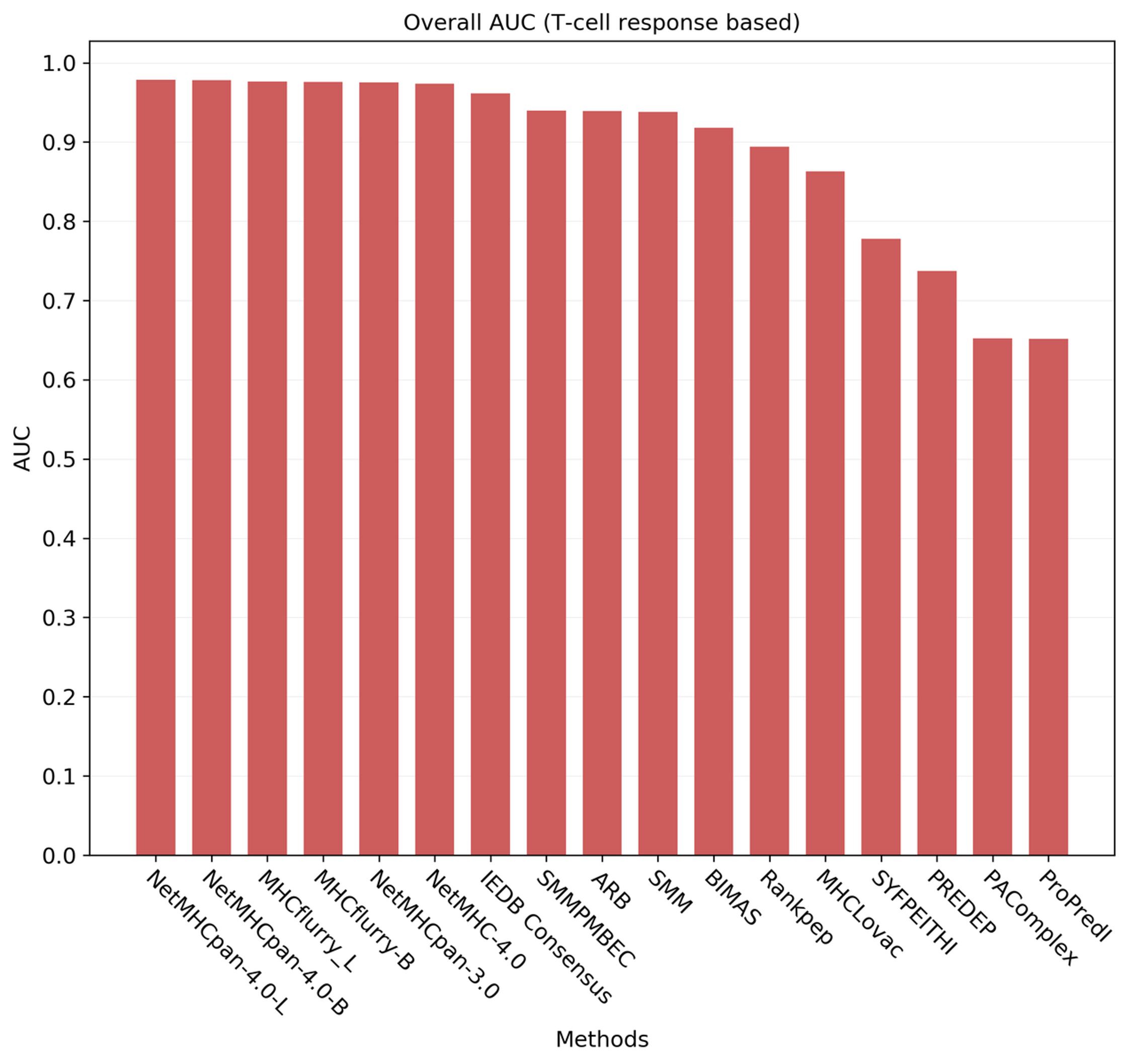

Supplement: S7 Fig — (TIF) [file pcbi.1007757.s008.tif]

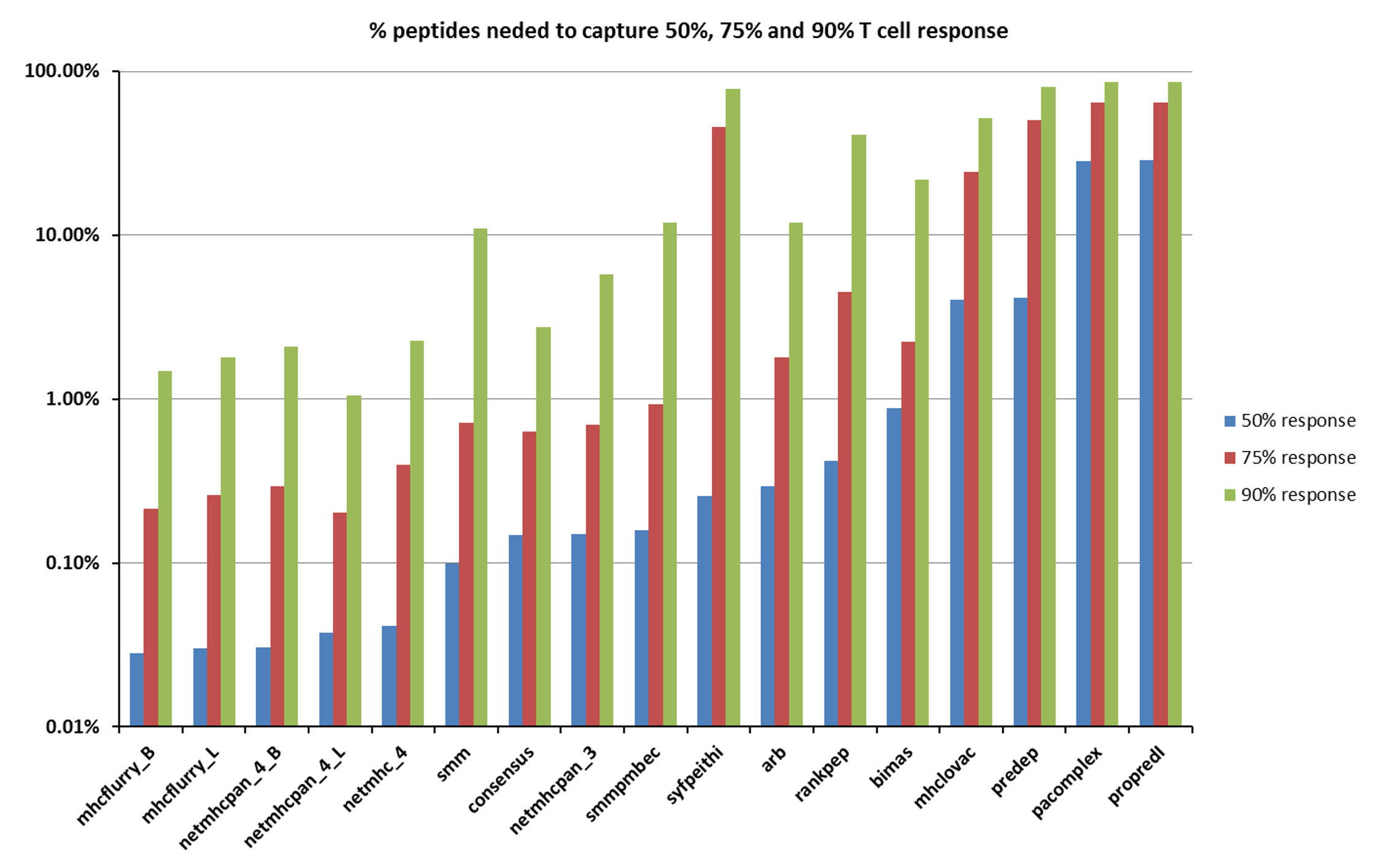

Supplement: S8 Fig — (TIF) [file pcbi.1007757.s009.tif]
